# Rift Valley Fever Virus Seroprevalence among Humans, Northern KwaZulu-Natal Province, South Africa, 2018–2019

**DOI:** 10.3201/eid2712.210643

**Published:** 2021-12

**Authors:** Janusz T. Pawęska, Veerle Msimang, Joe Kgaladi, Orienka Hellferscee, Jacqueline Weyer, Petrus Jansen van Vuren

**Affiliations:** National Institute for Communicable Diseases of the National Health Laboratory Service, Johannesburg, South Africa (J.T. Pawęska, V. Msimang, J. Kgaladi, O. Hellferscee, J. Weyer, P. Jansen van Vuren);; University of Pretoria, Pretoria, South Africa (J.T. Pawęska, J. Weyer);; Rand Water, Vereeniging, South Africa (J. Kgaladi);; Australian Centre for Disease Preparedness, CSIRO-Health and Biosecurity, Geelong, Victoria, Australia (P. Jansen van Vuren)

**Keywords:** Rift Valley fever virus, antibodies, transmission, endemic, cryptic, humans, mosquitoes, zoonoses, viruses, vector-borne infections, South Africa

## Abstract

We detected Rift Valley fever virus (RVFV) IgM and IgG in human serum samples collected during 2018–2019 in northern KwaZulu-Natal Province, South Africa. Our results show recent RVFV circulation and likely RVFV endemicity in this tropical coastal plain region of South Africa in the absence of apparent clinical disease.

Geographic expansion of Rift Valley fever virus (RVFV) associated with health and socioeconomic losses is of great concern for veterinary and public health professionals worldwide (*1*). In South Africa, major human Rift Valley fever (RVF) epidemics occurred in 1950–1951, 1974–1975, and 2010–2011 (*2*–*4*), but single outbreaks are reported only sporadically (*5*). RVF outbreaks in South Africa primarily have occurred on the temperate central plateau of the country (*6*), but historic data suggest circulation of RVFV in both humans and animals in the northern, tropical part of KwaZulu-Natal Province (*7*–*9*). Results of recent studies in this region show high RVFV seroprevalence in domestic goats (31.7%) and cattle (34%) (*10*) and in wild ruminants (35%) (*11*), without reported epizootics. To investigate the possibility of undetected RVFV infections in humans, we tested patients visiting healthcare facilities in northern KwaZulu-Natal for RVFV antibodies.

## The Study

Because of recent active circulation of RVFV in livestock and wildlife (*10*,*11*), we selected the uMkhanyakude Health District for active RVFV surveillance during April 2018–August 2019. Many households keep livestock composed of indigenous Nguni chickens, cattle, goats, or ducks. Participating locations 4 hospitals, Manguzi, Bethesda, and Mseleni, and Ndumo clinic attached to Mosvold hospital, and associated clinics, Mahlungulu, Makathini, and Mbazwana (Figure). The study was performed in accordance with protocols approved by the Human Research Ethics Committee of the University of Witwatersrand (Johannesburg, South Africa; approval nos. HREC M170606, M160667, and M161005) and provincial department of health (reference no. KZ_201709–037). 

**Figure:**
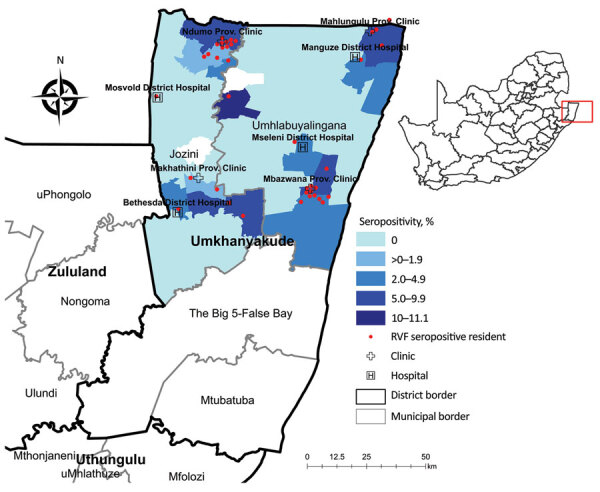
Distribution of human Rift Valley fever virus seropositivity and ward-specific seropositivity in northern municipalities of the uMkhanyakude District, KwaZulu-Natal Province, South Africa, April 2018–August 2019. Inset shows location of uMkhanyakude District (red box) in South Africa. Map was constructed in ArcGIS 10.2 (Esri, https://www.esri.com) using district, municipal, and ward boundaries, facilities, and participants’ residential coordinates collected during the study. Data are available under CC-BY 4.0 (Creative Commons Attribution, https://creativecommons.org) license.

Enrolled participants comprised persons >5 years of age of either sex who had measured axillary temperature of >37.5°C at examination or history of symptoms <7 days before examination, or at the time of examination, such as rash, headache, myalgia, arthralgia, and conjunctivitis. Study controls were persons from the same selected health facilities who were seeking healthcare for noninfectious conditions or for chronic care, and who had no history of fever <7 days. Case-controls were matched to age groups of enrolled participants as much as possible. Nurses conducted interviews and collected and recorded survey data on a case investigation form at the time the blood was drawn. Data were transferred into data gathering tool built on a tablet computer by using REDCap software (https://projectredcap.org), which is powered by Vanderbilt University (Nashville, Tennessee, USA). For analysis, we downloaded data from respective servers into Excel software (Microsoft, https://www.microsoft.com).

Nurses drew 5 mL of whole blood from participants 5–12 years of age and 10 mL from participants >12 years of age. Blood specimens were transported daily from clinics to their associated hospital laboratory for processing and temporary storage until transported for testing to the National Institute for Communicable Diseases of the National Health Laboratory Service (Johannesburg). We enrolled and collected samples from a total of 1,395 volunteers during April 2018–August 2019. 

We first tested serum samples by inhibition RVFV ELISA (*12*), then tested all positive samples by IgG sandwich ELISA and IgM capture ELISA, as previously described (*13*). We tested IgM-positive serum samples by using real-time reverse transcription PCR (rRT-PCR) (*14*). Of note, RVF and malaria can have similar clinical manifestations in patients, such as fever, arthralgia, and headache. Thus, we also tested specimens collected during April 2018–January 2019 for malaria antigen by using an ICT Malaria Combo Cassette Test (ICT International, https://www.ictdiagnostics.com), according to manufacturer instructions. We performed statistical analyses by using Stata version 13 (StataCorp LLC, https://www.stata.com) and Excel. We determined univariable statistics by using Fisher exact test for variables associated with RVFV seropositivity, such as sex, age, time outdoors, and agriculture activities. We used ArcGIS ArcMap 10.2 (Esri, https://www.esri.com) to create distribution and choropleth maps of RVF occurrence.

Among participants, 72.6% (997) were female and 27.4% (377) were male; no sex was recorded for 21 participants. The average age among participants was 35.3 (SD 17.0, range 5–96) years, and median was 33 (interquartile range 22–46) years. 

Of 1,395 volunteers tested, 39 tested RVFV positive by inhibition ELISA, of which 11 were positive for RVFV IgM and 9 for RVFV IgG (Table 1). The overall seropositivity adjusted for facility clustering was 2.8% (95% CI 1.45%–5.34%), and seropositivity differed significantly between facilities (p *=* 0.03) (Table 1). RVFV seropositivity was higher among groups >10 years of age compared with those 5–9 years old (p *=* 0.001) but was not significantly associated with sex (p *=* 0.481), spending time outdoors (p *=* 0.263), or working in agriculture (p *=* 0.161). None of the 11 IgM seropositive persons tested positive by RVFV rRT-PCR; 6 had fever at clinical examination at the healthcare facility. The most frequently observed symptoms were headache, myalgia, and arthralgia, and 3 participants had conjunctivitis (Table 2). Among IgM-positive participants, 3 were tested for malaria infection, and 2 tested positive. 

**Table 1:** Rift Valley fever virus IgG and IgM seropositivity in survey participants by healthcare facility and uMkhanyakude district, northern Kwazulu-Natal, South Africa, 2018–2019

Healthcare facility	No. tested	No. (%) seropositive*	No. (%) IgG positive†	No. (%) IgM positive‡
Mbazwana	185	8 (4.3)	7 (3.8)	1 (0.5)
Ndumo-Mosvold	377	16 (4.2)	14 (3.7)	7 (1.9)
Bethesda	178	5 (2.8)	5 (2.8)	1 (0.6)
Manguzi-Mahlungulu	207	5 (2.4)	5 (2.4)	1 (0.5)
Mseleni	178	4 (2.3)	4 (2.3)	1 (0.6)
Makhathini	270	1 (0.4)	1 (0.4)	0
Total	1,395	39 (2.8)	36 (2.6)	11 (0.8)

*Serum tested by an inhibition ELISA with 99.47% diagnostic sensitivity, 99.66% diagnostic specificity. This assay measures total Rift Valley fever virus antibody but does not discriminate between IgG and IgM (*12*). †Serum tested by an IgG-sandwich ELISA with 100% diagnostic sensitivity, 99.95% diagnostic specificity (*13*). ‡Serum tested by an IgM-capture ELISA with 96.47% diagnostic sensitivity, 99.44% diagnostic specificity (*13*).

**Table 2:** Symptoms and signs in Rift Valley fever virus in IgM-positive participants by health care facility, uMkhanyakude district, northern Kwazulu-Natal, South Africa, 2018–2019*

Healthcare facility	Age, y/sex	Fever	Rash	Headache	Myalgia	Arthralgia	Conjunctivitis	Vomiting	Malaria
Mbazwana	30/M	Y	N	Y	N	Y	Y	N	Y
Ndumo-Mosvold	55/F	N	N	Y	N	Y	N	N	N
	39/F	Y	Y	Y	Y	N	N	N	Y
	50/F	N	N	N	N	N	N	N	NT
	71/F	N	N	N	N	Y	N	N	NT
	27/M	Y	N	Y	Y	N	N	N	NT
	72/F	Y	N	Y	Y	Y	Y	N	NT
	25/F	N	N	Y	Y	Y	N	Y	NT
Bethesda	67/M	Y	N	N	Y	N	Y	N	NT
Manguzi-Mahlungulu	15/F	Y	N	N	N	Y	N	N	NT
Mseleni	47/F	N	N	Y	N	N	N	N	NT

*NT, not tested.

The east coast, the border with Mozambique, the Ndumo area in the north, Ubombo towards the south of the district where Bethesda is located, and the southeast near the iSimangaliso had higher RVFV seroprevalence, suggesting that more favorable conditions for RVFV circulation and human exposure exist in these areas. Of 11 IgM seropositive participants, 7 were seen in the Ndumo clinic, located in the northern section of the Jozini municipality and at the southern edge of Ndumo Game reserve and adjacent Tembe Elephant Park, part of the Lubombo Transfrontier Conservation and Resource area with Mozambique.

## Conclusions

Our serosurvey confirms recent exposure and indicates endemic circulation of RVFV in humans residing in the tropical coastal plain of northern KwaZulu-Natal Province in South Africa. The RVFV seropositivity we noted in our study is lower than that reported in the temperate inland of South Africa (*15*). The central plateau of South Africa is prone to RVF outbreaks, and more frequent and intense RVF outbreaks have occurred in the central plateau than the eastern coastal area (*6*). The inland of South Africa has the largest and most concentrated sheep farming regions. Sheep farms are not common in northern KwaZulu-Natal, and households keep livestock comprised mostly of indigenous cattle and goats. Among livestock, sheep, particularly newborn lambs, are most susceptible to RVFV infection (*1*,*6*). Most confirmed cases during the 2008–2011 RVF outbreak in South Africa were caused by physical contact with infected animals, either through disposal of dead animals or aborted fetuses, or slaughtering (*4*,*15*). No RVF outbreaks have been reported in northern KwaZulu-Natal, either in humans or animals, but recent findings suggest year-round virus transmission in cattle, goats (*10*), and wild antelopes (*11*) are associated with high RVFV seroconversion rates in domestic ruminants (*10*).

Study participants had detectable IgG and IgM to RVFV, and most IgM-positive samples were collected from participants with no recent history of travel beyond the study area. Our study indicates that RVFV infections in northern KwaZulu-Natal could be misdiagnosed or underreported, highlighting the urgent need for improved diagnostic testing and awareness of RVF and other arbovirus diseases in this part of South Africa. Moreover, our results suggest the possible role of the northern KwaZulu-Natal wildlife-livestock-vector host reservoir system in maintaining RVFV endemicity, including the potential to drive large-scale emergence and spread of the virus to other parts of the country. Because clinical manifestations of RVF in humans mimic those of malaria, RVFV surveillance can reduce potential misuse of antimalaria treatments. Our findings underscore the need for improved and active arbovirus biosurveillance in humans, wildlife, livestock, and mosquito vectors to mitigate associated transmission risk and potential RVF epidemics. 

## References

[1] Pawęska JT, Jansen van Vuren P. Rift Valley fever: a virus with potential for global emergence. In: Johnson N, editor. The role of animals in emerging viral diseases. London: Elsevier Academic Press; 2013. p. 169–200.

[2] Joubert JD, Ferguson AL, Gear J. Rift Valley fever in South Africa: 2. The occurrence of human cases in the Orange Free State, the north-western Cape province, the western and southern Transvaal. A Epidemiological and clinical findings. S Afr Med J. 1951;25:890–1. 14892941

[3] McIntosh BM, Russell D, dos Santos I, Gear JH. Rift Valley fever in humans in South Africa. S Afr Med J. 1980;58:803–6. 7192434

[4] Archer BN, Thomas J, Weyer J, Cengimbo A, Landoh DE, Jacobs C, et al. Epidemiologic investigations into outbreaks of Rift Valley fever in humans, South Africa, 2008–2011. Emerg Infect Dis. 2013;19:1918–25. 10.3201/eid1912.121527 29360021 PMC3840856

[5] Jansen van Vuren P, Kgaladi J, Msimang V, Pawęska JT. Rift Valley fever reemergence after 7 years of quiescence, South Africa, May 2018. Emerg Infect Dis. 2019;25:338–41. 10.3201/eid2502.181289 30666946 PMC6346436

[6] Swanepoel R, Coetzer JAW. Rift Valley fever. In: Coetzer JAW, Thomson GR, Tustin RC, editors. Infectious diseases of livestock with special reference to Southern Africa. Cape Town: Oxford University Press; 2004. p. 1, 688–717.

[7] Smithburn KC, Kokernot RH, Heymann CS, Weinbren MP, Zentkowsky D. Neutralizing antibodies for certain viruses in the sera of human beings residing in Northern Natal. S Afr Med J. 1959;33:555–61. 13675877

[8] McIntosh BM. Rift Valley fever. 1. Vector studies in the field. J S Afr Vet Med Assoc. 1972;43:391–5. 4670767

[9] McIntosh BM, Jupp PG, dos Santos I, Rowe AC. Field and laboratory evidence implicating *Culex zombaensis* and *Aedes circumluteolus* as vectors of Rift Valley fever virus in coastal South Africa. S Afr J Sci. 1983;79:61–4.

[10] van den Bergh C, Venter EH, Swanepoel R, Thompson PN. High seroconversion rate to Rift Valley fever virus in cattle and goats in far northern KwaZulu-Natal, South Africa, in the absence of reported outbreaks. PLoS Negl Trop Dis. 2019;13:e0007296. 10.1371/journal.pntd.0007296 31050673 PMC6519843

[11] Van den Bergh C, Venter EH, Swanepoel R, Hanekom CC, Thompson PN. Neutralizing antibodies against Rift Valley fever virus in wild antelope in far northern KwaZulu-Natal, South Africa, indicate recent virus circulation. Transbound Emerg Dis. 2020;67:1356–63. 10.1111/tbed.13479 31943795

[12] Pawęska JT, Mortimer E, Leman PA, Swanepoel R. An inhibition enzyme-linked immunosorbent assay for the detection of antibody to Rift Valley fever virus in humans, domestic and wild ruminants. J Virol Methods. 2005;127:10–8. 10.1016/j.jviromet.2005.02.008 15893560

[13] Pawęska JT, Burt FJ, Swanepoel R. Validation of IgG-sandwich and IgM-capture ELISA for the detection of antibody to Rift Valley fever virus in humans. J Virol Methods. 2005;124:173–81. 10.1016/j.jviromet.2004.11.020 15664066

[14] Drosten C, Göttig S, Schilling S, Asper M, Panning M, Schmitz H, et al. Rapid detection and quantification of RNA of Ebola and Marburg viruses, Lassa virus, Crimean-Congo hemorrhagic fever virus, Rift Valley fever virus, dengue virus, and yellow fever virus by real-time reverse transcription-PCR. J Clin Microbiol. 2002;40:2323–30. 10.1128/JCM.40.7.2323-2330.2002 12089242 PMC120575

[15] Msimang V, Thompson PN, Jansen van Vuren P, Tempia S, Cordel C, Kgaladi J, et al. Rift Valley fever virus exposure amongst farmers, farm workers, and veterinary professionals in central South Africa. Viruses. 2019;11:140. 10.3390/v11020140 30736488 PMC6409972

